# Construction and validation of an angiogenesis-related scoring model to predict prognosis, tumor immune microenvironment and therapeutic response in hepatocellular carcinoma

**DOI:** 10.3389/fimmu.2022.1013248

**Published:** 2022-11-17

**Authors:** Bo Tang, Xinyuan Zhang, Xiaozhou Yang, Wenling Wang, Rongkuan Li, Yu Liu

**Affiliations:** ^1^ Department of Hematology, The Second Affiliated Hospital of Dalian Medical University, Dalian, Liaoning, China; ^2^ Department of Infectious Disease, The Second Affiliated Hospital of Dalian Medical University, Dalian, Liaoning, China

**Keywords:** hepatocellular carcinoma, angiogenesis-related genes, prognosis, tumor immune environment, therapeutic response

## Abstract

**Background:**

Hepatocellular carcinoma (HCC) is one of the most common malignant tumors in the world with high morbidity and mortality. Identifying an effective marker for predicting the prognosis and therapeutic response is extremely meaningful. Angiogenesis-related genes (ARGs) play important roles in the tumor progression and immune-suppressive microenvironment formation.

**Methods:**

The differential expressed ARGs associated with the prognosis of HCC were identified in the TCGA dataset. Univariate Cox and least absolute shrinkage selection operator (LASSO) regression were applied to construct a ARGs Scoring model. The prognostic value of the ARGs Scoring model was assessed by Cox regression, Kaplan-Meier (KM) and ROC curve analyses. Then the model was further validated in an external dataset, ICGC dataset. The patients were split into two groups based on the ARGs Score and the clinical features were compared. TIMER, CIBERSORT and xCell algorithms were utilized to analyze the correlation between the ARGs Score and tumor immune microenvironment (TIME). Furthermore, we analyzed the efficacy of the model in predicting the therapeutic response for immunotherapy, targeted therapy and TACE treatment in different cohorts.

**Results:**

A total of 97 differential expressed ARGs were identified relating to the prognosis of HCC patients from the TCGA dataset. Then the ARGs Scoring model based on a 9-gene signature was constructed using the Cox and LASSO regression analyses. Higher ARGs Score had a poor clinical outcome and was considered to be an independent prognostic predictor for HCC in the multivariate Cox analysis. The ARGs Score was related to the enrichment of various immune cells, such as CD4+ T cells, Treg, macrophage, neutrophil and dendritic cells, exhibiting a more immunosuppressive phenotype. Higher ARGs Score was correlated with higher expression of immune checkpoint genes and poor response to immunotherapy. Furthermore, higher ARGs Score indicated poor therapeutic response in the sorafenib and TACE treatment cohorts, individually.

**Conclusions:**

The ARGs Scoring model exhibited robust predictive value for the prognosis and TIME for HCC patients. Higher ARGs Score indicated poor therapeutic response of the immunotherapy, sorafenib and TACE treatment. The ARGs Scoring model could be used as a biomarker to help physicians to develop more individualized treatment for HCC patients.

## Introduction

Primary liver cancer is the sixth most commonly diagnosed cancer with the third largest cancer mortality in the world in 2020 (1). The most common primary liver cancer is hepatocellular carcinoma (HCC) accounting for 90% of cases (2). The main risk factors for HCC are the infection by hepatitis B virus and hepatitis C virus. In addition, especially in the West, non-alcoholic steatohepatitis (NASH) is becoming the fastest increasing cause of HCC (3). There are some treatments for HCC including surgical resection, radiofrequency ablation, transcatheter arterial chemoembolization (TACE), targeted therapy, immunotherapy and liver transplantation (4). There are no specific clinical manifestations in the early stage of HCC. And owing to the lack of a parameter contributing to stratify the different stages, many patients are diagnosed at advanced stage and miss the best time for treatment. Only 10% of newly diagnosed HCC were considered ideal candidates for resection (5) and the major treatments for advanced HCC were targeted therapy and systemic chemotherapy (4). However, the overall survival (OS) has not improved significantly and nearly 70% HCC patients after surgery experience recurrence or extrahepatic metastasis within 5 years (6). Therefore, searching for new potential markers for prognostic prediction and therapeutic response is of important clinical significance to improve the prognosis of HCC patients.

Angiogenesis is a biological process that generates new vessels from the endothelium of existing vasculature for tissue growth, wound healing, and pregnancy (7, 8). Angiogenesis could supply oxygen and nutrients to the whole body, but on the other hand, it could nourish rapid growth and metastasis of tumor. The size of tumor cells could be no larger than 1–2 mm^3^ without angiogenesis due to hypoxia and poor nutrition (9). When the ratio of pro-angiogenic signals to anti-angiogenic signals increased, the endothelial cell proliferated and migrated to promote pathological angiogenesis making the tumor more aggressive. And angiogenesis is also an important immune evasion mechanism. Many evidences showed that sustained angiogenesis would lead to immune evasion through the induction of a highly suppressive tumor immune microenvironment (TIME) (10, 11). Angiogenesis is a crucial factor affecting the progression of HCC which is a typical hypervascular tumor. Drugs inhibiting angiogenesis such as sorafenib and lenvatinib are the first-line and systemic treatment for HCC patients (12). Immune checkpoint inhibitors (ICIs) could regulate and stimulate durable effective antitumor immune responses in many types of cancers including HCC (13, 14). And angiogenesis inhibitors combined with the ICIs could optimize the clinical outcome (15). However, sorafenib has a moderate antiangiogenic activity and some patients have to stop the therapy because of adverse effects or drug resistance (16). As to the ICIs, the immune-related adverse events can affect all organ systems and can be lethal in certain cases (17).

Hence, we systematically analyzed the expression of angiogenesis-related genes (ARGs) and explored the potential prognostic value for the prognosis and therapeutic response for HCC patients. A previous study demonstrated that ARGs signature could be used to predict the prognosis of HCC (18). In our study, we established a novel ARGs Scoring model based on 9 prognostic ARGs of HCC in The Cancer Genome Atlas (TCGA) dataset to predict the prognosis and validated this model in the International Cancer Genome Consortium (ICGC) dataset. Then we further analyzed the relationship of ARGs Score with the clinical features, TIME, drug sensitivity and therapeutic response of sorafenib and TACE treatment in HCC patients. These results indicated that the ARGs Scoring model could be utilized as a biomarker to predict the prognosis and therapeutic response in HCC. This study provided a novel tool that could be applied to help physicians to develop more individualized treatment for HCC patients.

## Materials and methods

**Figure 1:**
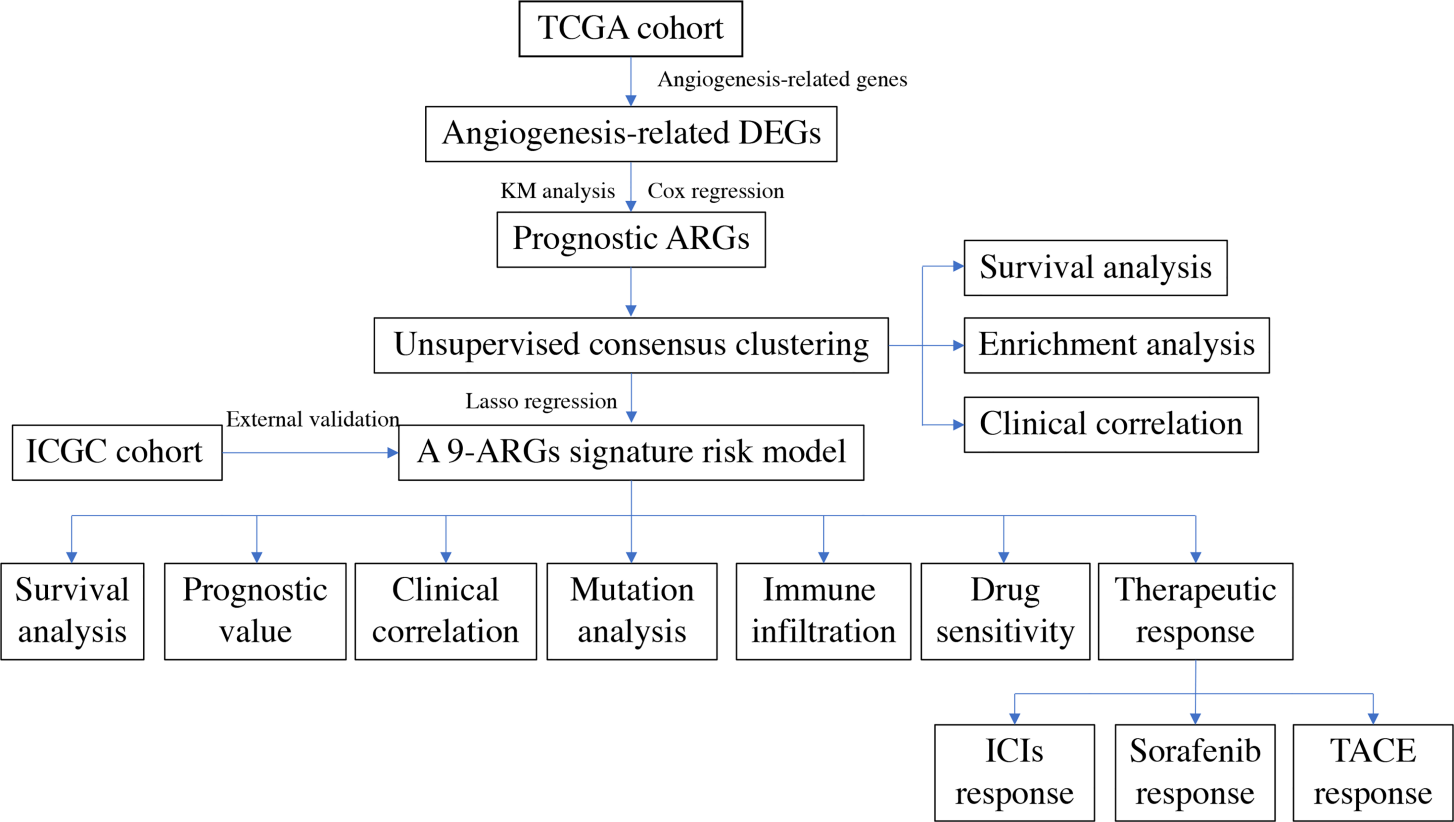
The flowchart of the entire study.

The flowchart of the entire study is shown in **Figure 1**.

### Data collection

The RNA sequencing dataset and corresponding clinical information were downloaded using UCSC Xena from TCGA dataset (https://portal.gdc.cancer.gov/) and ICGC dataset (https://dcc.icgc.org/). The TCGA-LIHC cohort containing 365 HCC samples with complete survival information was used as the training set, and the ICGC cohort containing 231 HCC samples was used as the validation set. The clinical features of the enrolled patients are detailed in **Table 1**. In addition, we obtained 67 HCC patients treated with sorafenib from GSE109211 cohort and 147 patients treated with TACE from GSE104580 cohort for therapeutic response analyses.

**Table 1:** Clinical features of the enrolled HCC patients.

Characteristics	TCGA cohort (n = 365)		ICGC cohort (N = 231)	
	N	%	N	%
Age (years)				
>65	138	37.81	142	61.5
≤65	227	62.19	89	38.5
Gender				
Male	246	67.4	170	73.6
Female	119	32.6	61	26.4
Grade				
G1	55	15.07		
G2	175	47.95		
G3	118	32.33		
G4	12	3.29		
T stage				
T1	180	49.32		
T2	91	24.93		
T3	78	21.37		
T4	13	3.56		
M				
M0	263	72.05		
M1	3	0.82		
N				
N0	248	67.95		
N1	4	1.1		
Stage				
Stage I	170	46.58	36	15.6
Stage II	84	23.01	105	45.5
Stage III	83	22.74	71	30.7
Stage IV	4	1.1	19	8.2

The ARGs were downloaded from the GeneCards and Molecular Signatures Database (MSigDB, http://www.broad.mit.edu/gsea/msigdb). A total of 1138 ARGs was obtained from the GeneCards database, and the screening criteria were protein coding genes and relevance scores greater than the median value (**Supplementary Table S1**). And 48 ARGs were downloaded from MSigDB (**Supplementary Table S2**).

### Identification of prognostic ARGs and functional enrichment analysis

The expression of ARGs between HCC and normal samples in the TCGA cohort was compared using R package “*limma*”. A false discovery rate of p value<0.05 and |log2FC | >1 were considered as statistically significant. Univariate Cox proportional hazards regression and Kaplan-Meier (KM) analyses were performed using the “*survival*” R package to screen ARGs associated with OS. p-value less than 0.05 was selected for further analysis. Gene Ontology (GO) and Kyoto Encyclopedia of Genes and Genomes (KEGG) enrichment analyses were performed with the “*clusterProfiler*” R package.

### Consensus clustering analysis of ARGs

HCC patients from TCGA-LIHC dataset were clustered into distinct subtypes using the “*ConsensusClusterPlus*” package in R software according to the expression of the prognostic ARGs with the parameters of 1000 iterations, resample rate of 0.8. The optimal number of clusters was determined when the cumulative distribution function (CDF) curve in the range of 0.1–0.9 was near flat. The heatmap was applied to show the correlation between clusters and clinical features. The OS analysis of different clusters was evaluated by the KM plot.

### Construction and validation of the ARGs scoring model

To screen the most relevant ARGs with the prognosis of HCC patients, the least absolute shrinkage and selection operator (LASSO) analysis with the “*glmnet*” package was used to build a prognostic model to avoid overfitting (19). The candidate genes that constituted the prognostic model and their coefficients were consequently identified through the optimal penalty parameter λ associated with the smallest 10-fold cross validation. Then the ARGs Score for each sample was calculated according to the following formula: *ARGs* *score*=∑(*Expi* * *coefi*) , where Expi represents the candidate gene expression level and coefi represents the corresponding coefficient. HCC patients in the training and validation cohorts were divided into high-score and low-score group according to the median ARGs score. Principal component analysis (PCA) was performed by the “*prcomp*” function in the “*stats*” R package to validate the reliability of clustering based on the ARGs Score. OS analysis based on the KM curve was conducted between the two groups. To study the predictive ability of the ARGs Scoring model over time, the “*TimeROC*” R package was used to present the time-dependent receiver operating characteristic (ROC) curve.

The differences of clinical features between high-score and low-score groups were analyzed by Wilcoxon signed-rank test and Chi-square test in the TCGA dataset. To verify the independency of the ARGs Score as a prognostic predictor, the univariate and multivariate Cox analysis was performed in the training and validation cohorts.

### Analysis of tumor somatic mutations and tumor mutation burden

The tumor somatic mutations data from TCGA dataset was analyzed between high-score and low-score groups by the “*maftools*” R package. The top 20 mutation genes were obtained and then compared between ARGs Score subgroups. Tumor mutation burden (TMB) was defined as the total number of somatic mutations per megabase in each tumor sample. The TMB of each sample was calculated and then compared between the high-score and low-score groups. The prognostic value of TMB was evaluated by the KM plot.

### Immune landscape analysis

The infiltrating immune cells levels were calculated by TIMER (20), CIBERSORT (21) and xCell (22) algorithms in each HCC sample and compared between the high-score and low-score groups. ESTIMATE algorithm was applied to perform the calculation of the immune score, stromal score and estimate score based on the proportion of immune cells and stromal cells (23).

### Evaluation of the immunotherapy efficacy and drug sensitivity analysis

The Tumor Immune Dysfunction and Exclusion (TIDE) algorithm (24) is utilized to model tumor immune evasion and has been applied in many studies to evaluate immunotherapy efficacy. And the immunophenoscore (IPS) from The Cancer Immunome Atlas (TCIA) database (https://tcia.at/) was used to predict the immunotherapy response (anti-PD-1 and anti-CTLA4) as described previously. The TIDE and IPS were compared between the high-score and low-score groups to predict immunotherapy efficacy to ICIs. We further utilized the “*oncoPredict*” R package to assess the chemotherapeutic response by predicting the half-maximal inhibitory concentration (IC50) based on Genomics of Drug Sensitivity in Cancer (GDSC) (https://www.cancerrxgene.org/). The estimated IC50 was compared between the different ARGs Score groups.

### Statistical analysis

All statistical analyses were performed using R version 4.1.1 and various R packages. The Chi-squared test was applied to compare the constituent ratio of two subgroups. Continuous variables in two or more groups were compared using Wilcoxon rank-sum test or Kruskal–Wallis test. The correlation between two continuous variables was measured by Spearman correlation analysis. Univariate and multivariate Cox regression analysis was utilized to confirm the independent prognostic value of the ARGs Score. All statistical p-values were two-sided, with p< 0.05 considered as statistically significant.

## Results

### Identification of prognostic differentially expressed ARGs and functional enrichment analysis

**Figure 2:**
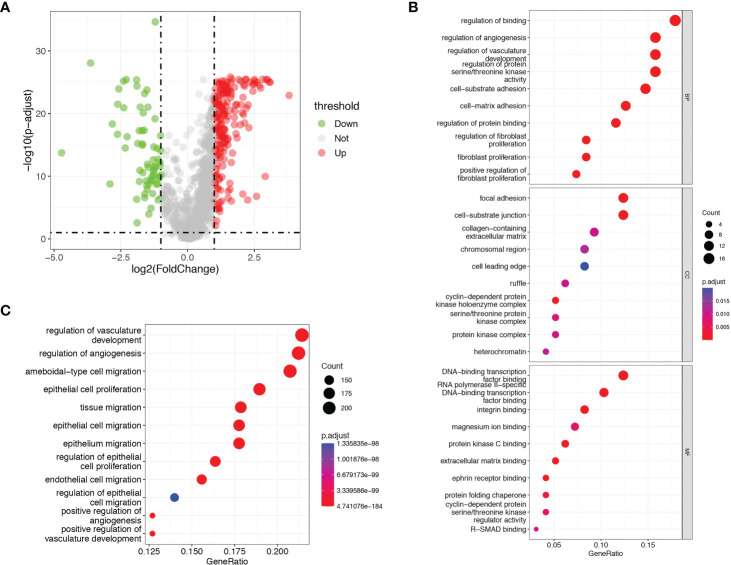
Identification of prognostic differentially expressed ARGs. **(A)** Volcano plots showed the prognostic differentially expressed ARGs in TCGA dataset. **(B)** GO analysis of the identified ARGs. **(C)** KEGG analysis of the identified ARGs.

A total of 1146 ARGs from GeneCards database and MSigDB were enrolled for analysis in our study. We obtained 249 differentially expressed ARGs between tumor and normal tissues in TCGA-LIHC dataset according to the threshold of |log2FC| ≥1 and p< 0.05 (**Figure 2A** and **Supplementary Table S3**). Next, we sought to evaluate the predictive value of ARGs for prognosis in HCC. Univariate Cox proportional hazards regression and KM analysis were employed to screen the prognostic genes in the differentially expressed ARGs in HCC patients. Finally, a total of 97 ARGs were considered to be associated to the OS of HCC patients (**Figure S1** and **Supplementary Table S4**).

GO and KEGG pathway enrichment analyses were performed with a cut-off of p< 0.05. GO analysis showed that the identified prognostic ARGs were mainly enriched in regulation of binding, regulation of angiogenesis, regulation of vasculature development and regulation of protein serine/threonine kinase activity (**Figure 2B**). KEGG pathway analyses showed that the identified prognostic ARGs were mainly involved in the angiogenesis regulating and epithelial cell proliferation/migration, such as regulation of vasculature development, regulation of angiogenesis, ameboidal−type cell migration and epithelial cell proliferation (**Figure 2C**). These enrichment analyses suggested that the identified ARGs were closely related to the angiogenesis regulation and pathways.

### Molecular clustering based on the prognostic ARGs

**Figure 3:**
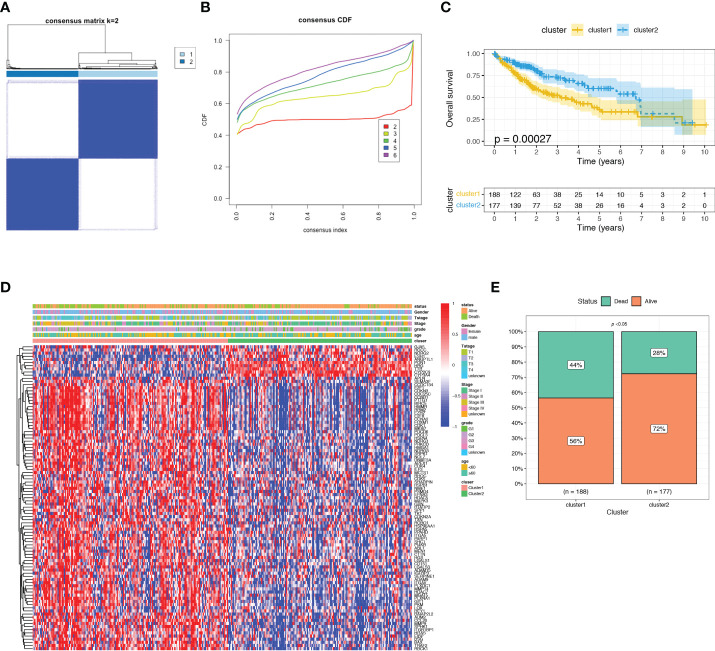
Consensus clustering analysis of identified prognostic ARGs in TCGA dataset. **(A)** Consensus matrix heatmap defining two clusters (k = 2). **(B)** Consensus clustering cumulative distribution function (CDF) with k valued 2 to 9 in TCGA dataset. **(C)** KM curve for the two clusters. **(D)** Differences in clinical characteristics and ARGs expressions between the two distinct clusters. **(E)** The surviving fraction compared between Cluster 1 and Cluster 2.

In order to investigate the relationship between the expression of ARGs and prognosis of HCC, a consensus clustering analysis was performed in TCGA-LIHC dataset. The results of consensus clustering suggested that 365 HCC patients could be divided into two clusters according to the expression of the identified prognostic ARGs, and the optimal number of clusters (k = 2) was determined by CDF curve (**Figures 3A–B** and **Figure S2**). KM analysis showed that Cluster1 was significantly correlated with a worse OS than the Cluster2 (**Figure 3C**). The heatmap showed that the clusters were correlated with the expression of prognostic ARGs, 10 genes were upregulated in Cluster2 while the rest 87 genes were upregulated in the Cluster1 (**Figure 3D**). Then we investigated the clinical features of the two clusters in the TCGA-LIHC dataset. The Cluster1 was correlated with a higher histological grade, T stage and clinical stage (**Figure S3A–E**). The surviving fraction was higher in Cluster2 than in Cluster1 (**Figure 3E**).

### Construction of a prognostic ARGs scoring model

**Figure 4:**
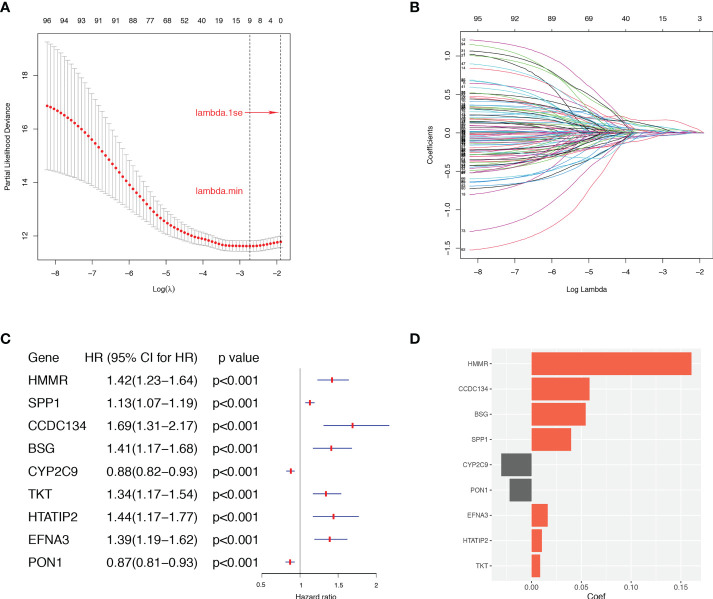
The LASSO regression analysis in the TCGA dataset. **(A)** Selection of the optimal parameter (lambda.min) in the LASSO model. **(B)** LASSO coefficient profiles of ARGs in TCGA dataset. **(C)** The retained 9 candidate genes. **(D)** LASSO coefficient of the 9 candidate genes for ARGs Scoring model construction.

**Figure 5:**
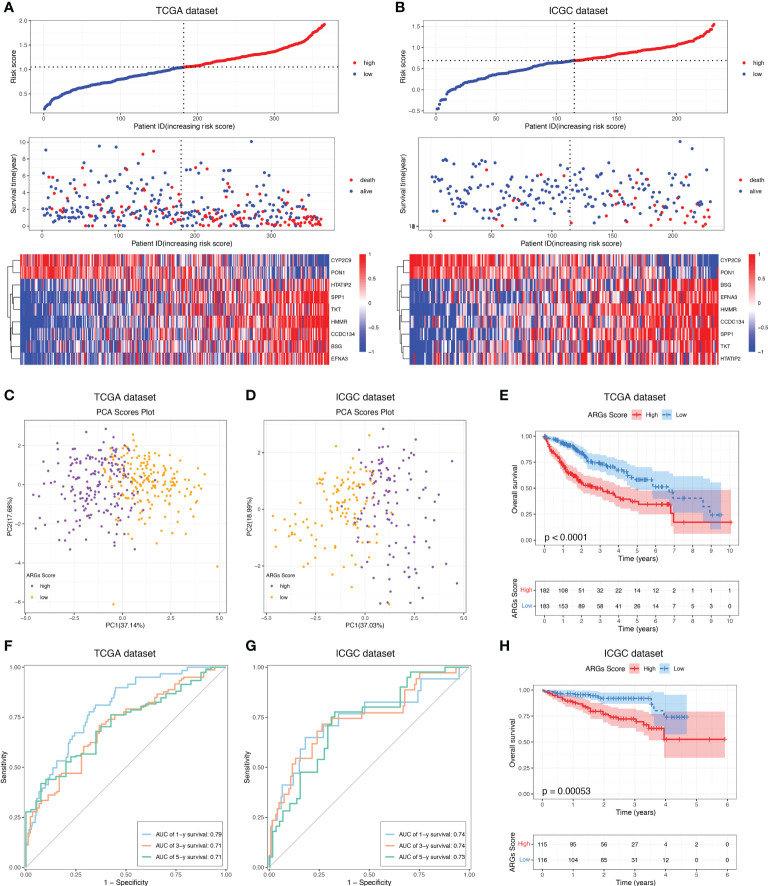
Construction and validation of the ARGs Scoring model. The distribution of the ARGs Score, survival status and ARGs expression of HCC patients in the **(A)** TCGA and **(B)** ICGC cohorts. PCA analysis of the HCC patients based on the ARGs Score in the **(C)** TCGA and **(D)** ICGC cohorts. KM analyses of the ARGs Score in the **(E)** TCGA and **(H)** ICGC cohorts. Time-dependent ROC curve of the ARGs Score in the **(F)** TCGA and **(G)** ICGC cohorts.

To construct a more applicable classifier to predict the prognosis of HCC patients, we performed LASSO regression analysis of the 97 prognostic ARGs to remove redundant genes and avoid overfitting problems. Lambda.min exhibited minimum partial likelihood deviance and was considered as the optimal λ for fitting the model (**Figure 4A**). Each curve corresponds to a gene and the vertical axis represents the coefficient of each gene (**Figure 4B**). As a result, 9 signature ARGs were retained (**Figure 4C**) and the model coefficients could be calculated at the value of Lambda.min (**Figure 4D**). Of them, PON1 and CYP2C9 were protective genes for HCC survival with HR<1, and HMMR, SPP1, CCDC134, HTATIP2, BSG, TKT and EFNA3 were risk genes with HR>1 (**Figure 4C** and **Supplementary Table S4**). The ARGs Score of each HCC patient was calculated using the gene expression profiles and estimated regression coefficients according to the formula mentioned in the method. Then HCC patients in the training cohort, TCGA-LIHC, were divided into a low-score group and a high-score group according to the median ARGs Score. The risk plot showed that the mortality rate in the training cohort increased with the increasing ARGs Score (**Figure 5A**). The PCA analysis further validated that HCC patients could be well separated into two groups based on the ARGs Score (**Figure 5C**). KM survival curve indicated that high-score HCC patients had poorer prognosis than low-score patients (**Figure 5E**). Then this ARGs Scoring model was evaluated by time-dependent ROC analysis. The area under the ROC curve (AUC) for 1-, 3- and 5-year OS in the TCGA dataset were 0.79, 0.71 and 0.71, respectively (**Figure 5F**). These results indicated that the ARGs Score had good predictive accuracy for prognosis of HCC in the TCGA cohort.

### Validation of the ARGs scoring model

To demonstrate the predictive value of the ARGs Scoring Model, the ICGC dataset was employed as the validation cohort. The ARGs Score of each patient was calculated according to the same formula in the training cohort and the patients were then assigned into two groups depending on the median of ARGs Score. The risk plot presented a clear separation of survival status between the two groups and the red dots represented deceased patients and blue ones alive patients (**Figure 5B**). Similarly, PCA analysis showed a clear distribution between the two groups (**Figure 5D**) and high-score group was significantly correlated to a poorer prognosis than the low-score group (**Figure 5H**). Besides, the ROC analysis of the ARGs Scoring Model in the validation cohort showed that the AUCs was 0.74 in 1-year, 0.74 in 2-year, and 0.73 in 3-year (**Figure 5G**), suggesting that the ARGs Scoring Model had an excellent predictive value in the prognosis of HCC patients.

### Analysis of the correlation between ARGs score and clinical features

**Figure 6:**
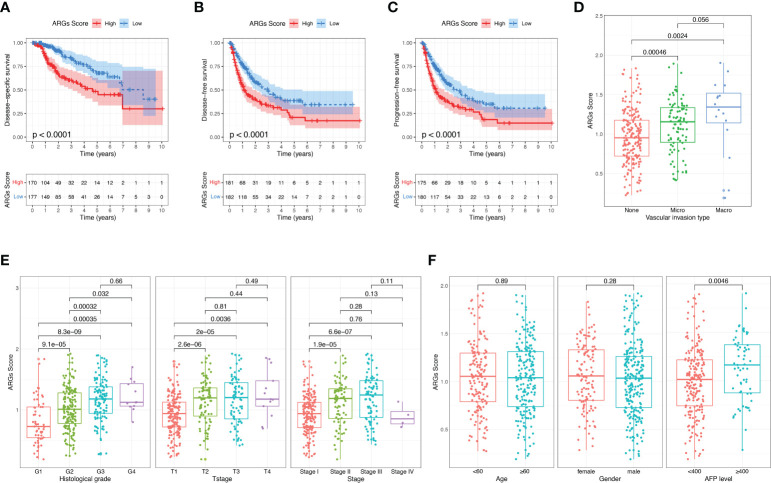
Correlation between the ARGs Score and clinical features in TCGA dataset. **(A–C)** The disease-free survival, disease-specific survival and progression-free survival analyses between the high-score and low-score groups. **(D)** The relationship between the ARGs Score and vascular invasion type. **(E, F)** Correlation of the ARGs Score with clinical stage, T stage, histological grade, age, gender and AFP level.

Then we investigated the correlations between the ARGs Score and clinicopathological features of HCC patients in TCGA-LIHC dataset. The high-score group had a markedly worse disease-free survival, disease-specific survival and progression-free survival than the low-score group (**Figures 6A–C**). The ARGs Score was much higher in the patients with vascular invasion, especially in the patients with macrovascular invasion (**Figure 6D**). Increased ARGs Score was significantly associated with the progression of HCC, such as the advanced histological grade, clinical stage and T stage (**Figure 6E**). The ARGs Score was significantly positively correlated with the AFP level, but did not vary with the age and gender (**Figure 6F**).

**Figure 7:**
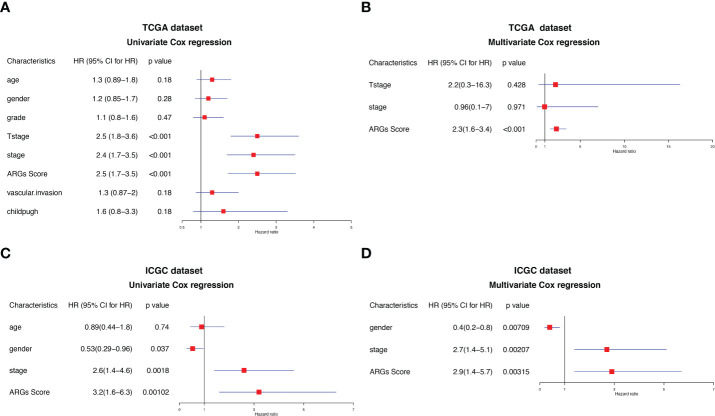
Univariate and multivariate Cox regression analysis of the ARGs Score. Univariate Cox regression analysis in the **(A)** TCGA and **(C)** ICGC cohorts. Multivariate Cox regression analysis in the **(B)** TCGA and **(D)** ICGC cohorts.

To further determine the independency of the ARGs Score as a prognostic predictor for OS in HCC patients, univariate and multivariate Cox regression was conducted in the TCGA-LIHC and ICGC datasets, individually. In univariate Cox regression analysis, the clinical stage, T stage and ARGs Score were significantly associated with OS in the TCGA-LIHC cohort (**Figure 7A**). After correction for other confounding factors, multivariate Cox regression analysis further confirmed that ARGs Score was an independent prognostic factor in HCC (**Figure 7B**). The results were verified in the ICGC cohort (**Figures 7C–D**).

### Analysis of the correlation between ARGs score and mutation landscape

**Figure 8:**
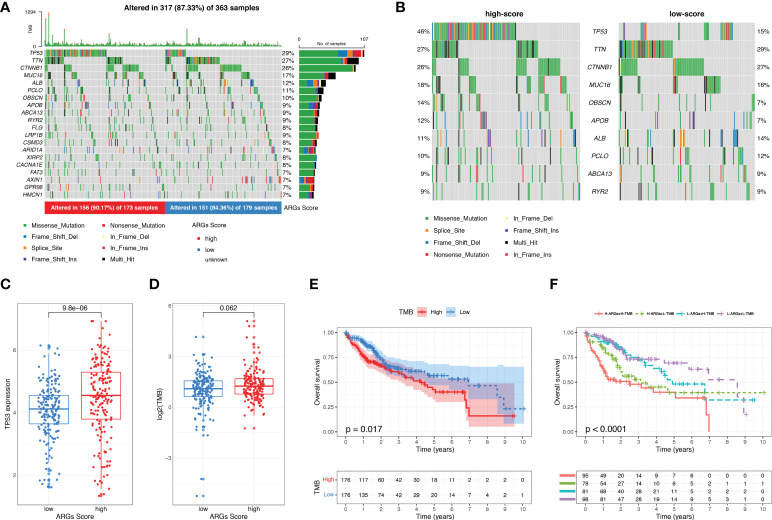
The mutation landscape and tumor mutation burden analysis. **(A)** Waterfall plots about the mutation distribution of the top 20 most frequently mutated genes in HCC patients from the TCGA dataset. **(B)** Comparison of the top 10 mutation genes between low-score and high-score groups. **(C)** The expression of TP53 between low-score and high-score groups. **(D)** TMB between low-score and high-score groups. **(E)** KM analysis of HCC patients stratified by TMB. **(F)** The survival analysis of HCC patients based on the combination of ARGs Score and TMB.

We next investigated the tumor somatic mutations between different ARGs Score groups based on TCGA dataset. The top 20 variant mutations in the TCGA-LIHC cohort were identified and a higher fraction of patients with mutated genes appeared in the high-score group than the low-score group (90.17% vs. 84.36%, **Figure 8A**). A much higher TP53 mutation frequency existed in the high-score group than the low-score group (46% vs. 15%, **Figure 8B**). And the TP53 expression was higher in the high-score group (**Figure 8C**). TMB is attributed to genomic instability and is considered as a predictor of the immunotherapy in various tumors. We calculated the TMB for each HCC patient. TMB was slightly higher in the high-score group than in the low-score group (p=0.074, **Figure 8D**) and the higher TMB was related to the poor prognosis of HCC (**Figure 8E**), suggesting that the ARGs Score can also reflect the level of tumor mutation burden. The combination analysis of ARGs Score and TMB showed that the high ARGs Score and high TMB were related to the poor prognosis of HCC (**Figure 8F**).

### Analysis of the correlation between ARGs score and immune landscape

**Figure 9:**
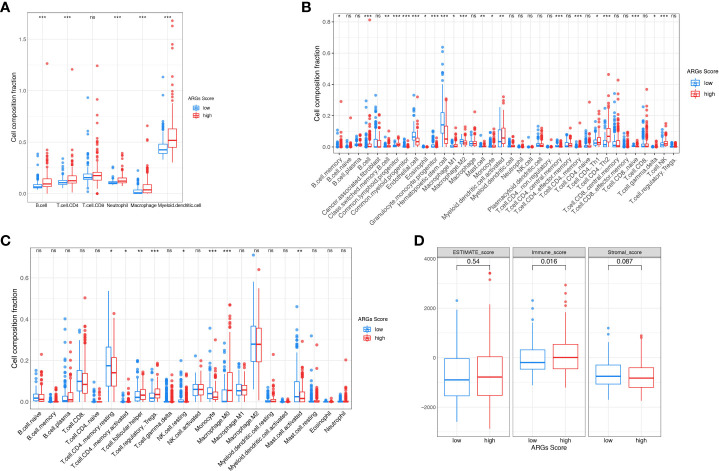
The landscape of tumor immune microenvironment. The composition of tumor infiltration immune cells was compared between high-score and low-score groups based on the TIMER **(A)**, CIBERSORT **(B)** and xCell **(C)**. **(D)** Stromal score, Immune score and ESTIMATE score between high-score and low-score groups in TCGA. *p< 0.05; **p< 0.01; ***p< 0.001. ns, not significant.

To further explore the potential correlation between the ARGs Score and the immune landscape of HCC, we consequently evaluated immune infiltration between the high-score group and low-score group. In the TIMER algorithm, the B cell, CD4^+^ T cell, neutrophil, macrophage and myeloid dendritic cell were much higher in the high-score group (**Figure 9A**). While more subgroups of immune cells were analyzed with CIBERSORT and xCell algorithm, the B cell, Th1 cell, Th2 cell, Treg cell, NKT cell and macrophages were higher in the high-score group (**Figures 9B–C**). Then the immune score, stromal score, and ESTIMATE score were analyzed and compared between the low-score and high-score group using the ESTIMATE algorithm (**Figure 9D**). The immune score was significantly higher in the high-score group than the low-score group, implying that the immune cells were abundant in the high-score group. There was no difference of stromal score and ESTIMATE score between the two groups, indicating that the stromal cells and tumor purity were not significantly associated with the ARGs Score.

### The prognostic value of the ARGs score in the prediction of therapeutic response

**Figure 10:**
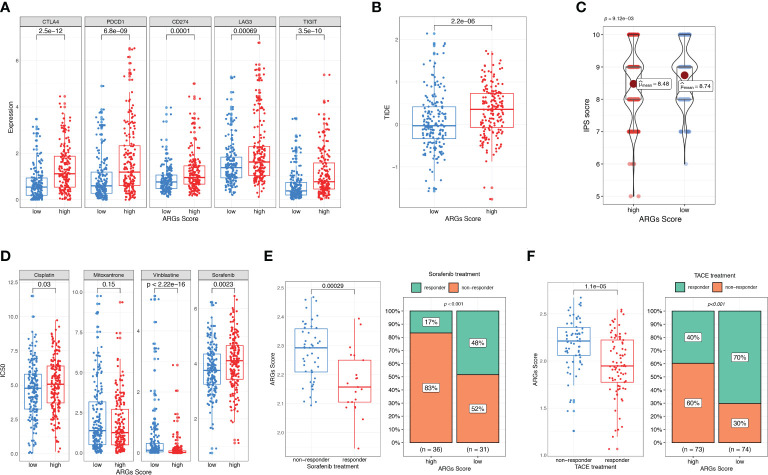
Evaluation of therapeutic response and drug sensitivity based on the ARGs Scoring model. **(A)** The correlation between ARGs Score and immune checkpoints genes. Difference in TIDE **(B)** and IPS **(C)** between the high-score and low-score groups. **(D)** Drug sensitivity analysis. IC50 of different drugs were compared between high-score and low-score groups. **(E)** Comparison of ARGs Score between sorafenib responder and non-responder. The fraction of sorafenib responder between subgroups based on ARGs Score classifier. **(F)** Comparison of ARGs Score between TACE responder and non-responder. The fraction of TACE responder between subgroups based on ARGs Score classifier.

As the ICIs are widely used in clinical treatment, we further investigated the relationship between ARGs Score and the expressions of immune checkpoints, such as CTLA-4, TIGIT, PD-1, PD-L1 and LAG3. We found that these genes were significantly up-regulated in the high-score group (**Figure 10A**). TIDE and IPS are widely used to evaluate the immunotherapy response of ICIs. And higher TIDE and lower IPS suggest more effective response to immunotherapy. To better illustrate the predictive value of the ARGs Score for immunotherapy, we applicated TIDE and IPS in the TCGA cohort. The patients in the high-score group had higher TIDE score and lower IPS score (**Figures 10B–C**), indicating that HCC patients with higher ARGs Score had more immune dysfunction and poorly respond to immunotherapy. Next, we estimated the IC50 values of drugs to explore if the ARGs Score was related to the drug sensitivity. Patients in the low-score group were significantly more sensitive to cisplatin, vinblastine and sorafenib (**Figure 10D**).

To further validate the predictive value of ARGs Score for other treatments, we analyzed the ARGs Score for HCC patients who received sorafenib and TACE treatment. In the sorafenib treatment cohort, patients who achieved a significant extended recurrence-free survival were considered as the responders (25). The ARGs Score was calculated and compared between the two groups. As shown in the **Figure 10E**, the ARGs Score was significantly higher in the non-responders than the responders. Compared to the patients in the low-score group, there was a much lower fraction of sorafenib responders in the high-score group (17% vs. 48%, p<0.001), indicating that the higher ARGs Score was a valuable predictor for poor response with sorafenib. We further explored the predictive power of the ARGs Scoring model in a TACE treatment cohort. The evaluation of the response to TACE was assessed by extramural reviewers using the modified *Response Evaluation Criteria in Solid Tumors (* 26). The patients with a complete response or a partial response were grouped as having an objective response to TACE, whereas patients with stable disease or progressive disease were grouped as having non-response to TACE. Similar to the results in sorafenib treatment cohort, the ARGs Score was higher in the high-score group and the percentage of responders was much higher in the low-score group (70% vs. 40%, **Figure 10F**).

## Discussion

HCC is a leading cause of cancer-related death in many areas of the world. Although the measures of prevention, surveillance, early detection, diagnosis and treatment have been widely implemented, the incidence and mortality of HCC continue to increase in many countries. Compared with the decreasing disease burden of many other major cancers, the overall burden of HCC worldwide is still increasing over time (27). Angiogenesis is the process of generating new capillaries regulated by angiogenic and anti-angiogenic factors. Angiogenesis can provide adequate oxygen and nutrients for tumor cells contributing to the tumorigenesis, metastasis, and migration. HCC is a highly hypervascularised tumor and characterized by hypoxia which promotes the tumor growth and progression (28). Several pro-angiogenic growth factors such as vascular endothelial growth factor (VEGF), platelet-derived growth factor (PDGF), insulin-like growth factor-1 (IGF-1), transforming growth factor (TGFβ) and fibroblast growth factor (FGF) are overexpressed in HCC patients (16). Anti-angiogenic agents such as tyrosine-kinase inhibitors (TKI) and VEGF inhibitors, targeting various pro-angiogenic signaling pathways have been validated to improve survival in advanced HCC (12).

In our study, we identified 249 differentially expressed ARGs in HCC samples from the TCGA dataset, and 97 of them were significantly associated with the prognosis. The identified prognostic ARGs were mainly involved in the biological processes of angiogenesis, such as regulation of angiogenesis, regulation of vasculature development and epithelial cell proliferation. Then an ARGs Scoring model was constructed based on a 9-gene signature using the Cox and LASSO regression. HCC patients could be classified into high-score and low-score groups according to the ARGs Score. The survival analysis demonstrated that the higher ARGs score was significantly associated with the poor prognosis of HCC, including OS, disease-free survival, disease-specific survival and progression-free survival. And the PCA analysis further validated the ARGs Score could be used as a good classifier for HCC patients. The ARGs Score was proved as an independent prognostic factor for the clinical outcome. The predictive ability of the ARGs Score was determined by the time-dependent ROC curve, showing a relatively robust diagnostic value in predicting 1-year survival (AUC = 0.79) and 5-year survival (AUC = 0.71). Besides, the ARGs Scoring model was further demonstrated in the validation cohort, ICGC dataset. And we observed that higher ARGs Score significantly suggested more remarkable progression of clinical stages, histological grades and T stages in HCC patients. ARGs Score was also positively correlated with severe vascular invasion. These results indicated that the ARGs Scoring model could be utilized as a prognostic biomarker for HCC patients.

The TIME of HCC is a complex mixture of hepatic non-parenchymal resident cells, tumor cells, immune cells and tumor-associated fibroblasts (29). The interplay between the tumor cells and TIME plays important roles in tumor initiation, progression, metastasis and response to therapies. The adaptive immune response in patients with HCC is weakened with exhaustion or dysfunction of tumor-infiltrating lymphocytes, particularly CD4+ and CD8+ T cells (30). CD8+ T cells, as the cytotoxic T lymphocytes that can recognize tumor-associated antigens and then destroy tumor cells, have significant correlation to OS (31). The subsets of CD4+ T cells include Th1, Th2, Th17, Treg cells and Tfh cells. Treg cells increase in HCC and the function of CD8+ T cells are impaired by Treg cells resulting in the promotion of tumor evasion and disease progression (32). Some evidences showed that Th1, Th2 and Treg cells make contributions to angiogenesis *via* various mechanisms (11) (33). The immunosuppressive cells of HCC mainly include tumor-associated macrophages (TAMs), tumor-associated neutrophils (TANs), DCs and Treg cells. In our study, the CD8+ T cells were slightly higher in the low-score group which had a better survival than the high-score group. And the higher ARGs Score was positively correlated to the enrichment of Th1, Th2, Treg, TAMs, DCs and TANs. Thus, the ARGs Score could reflect the TIME in HCC and exhibited a more suppressive immune phenotype. However, the immune cell composition was calculated based on the various algorithm and it is still inaccurate compared with immunohistochemistry and flow cytometry. Even so, the TIME analysis could still contribute to learn more about tumor immunity. Overall, the ARGs Score was significantly correlated with tumor infiltrating cells indicating that it might contribute to the immune regulation involving in the progression of HCC.

As the inhibitory immunoreceptors expressed by effector immune cells, immune checkpoints can restraint the overactivation of the effector immune cells. This physiological mechanism is utilized by HCC and other solid tumors to avoid anti-tumor immune responses with the expression of the corresponding ligands in tumor and stromal cells (34). CTLA4 is mainly expressed on the surface of activated T cells and Treg cells regulating T cell tolerance (35). PD-1 is expressed on various immune cells, including the activated T cells, NK cells, Treg, MDSCs, monocytes and DCs. It can bind to its ligands PD-L1 and PD-L2 which are expressed in many tumors including HCC, transmitting inhibitory signals to T cells and resulting in the immune evasion of tumor cells. ICIs targeting PD1, PD-L1, and CTLA-4 are the most commonly used immunotherapy in the field of advanced HCC. Various immune-related adverse events, such as pneumonitis, myocarditis, hypophysitis, diabetes and so on, can be induced by ICIs, limiting the widely application for many patients in need. Therefore, it is very important to predict the therapeutic effect and balance the benefits and adverse effects for patients receiving the ICIs treatment. In our study, we evaluated the relationship of the ARGs Score with the immunotherapy efficacy. The results showed that the ARGs Score was significantly positively correlated with the expression of immune checkpoints, CTLA4, PD1 and PD-L1. TIDE and IPS have been applied in many studies to evaluate immunotherapy response of ICIs. Our work showed that the ARGs Score was presenting a similar trend in predicting the immunotherapy response as the TIDE and IPS. These results indicated that the ARGs Score could be employed as a robust predictor of immunotherapy response for HCC patients.

Many patients were initially diagnosed with unresectable HCC. As the first drug of first-line systemic treatment for patients with advanced-stage HCC, sorafenib has been shown to extend the survival of patients (36). TACE has been commonly used as the standard treatment for the intermediate-stage HCC patients (37). The response rate was about 52.4% and the major adverse events included the liver enzyme abnormalities, fever and haematological or bone marrow toxicity (38). In our study, the drug sensitivity analysis found that the IC50 value of sorafenib was lower in the low-score group indicating that patients with low ARGs Score might had a better response to sorafenib treatment. Then in the sorafenib treatment cohort, more sorafenib responders existed in the low-score group which means that the ARGs Score could be used as a predictive marker for sorafenib treatment response. The similar results were also observed in the TACE treatment cohort. These results showed that the ARGs Score is of great clinical significance for predicting the therapeutic responses for HCC patients.

Taken together, we conducted a relatively comprehensive study and constructed an ARGs Scoring model to predict the prognosis and therapeutic response for HCC patients. Meanwhile, we recognized some limitations in this study. Firstly, the ARGs Scoring model was constructed and validated based on the gene expression files from the public datasets and required to be further verified through prospective cohort studies. Secondly, we need more independent immunotherapy cohorts to verify the predictive value of ARGs Score for immunotherapy efficacy. Lastly, further experimental validation is needed in order to confirm these observations in the future.

## Conclusion

We constructed an ARGs Scoring model for predicting the prognosis and TIME of HCC with high accuracy. Higher ARGs Score indicated poor therapeutic response of the immunotherapy, sorafenib and TACE treatment. The ARGs Scoring model could be used as a biomarker to help physicians to develop more individualized treatment for HCC patients.

## Data availability statement

The original contributions presented in the study are included in the article/**Supplementary Material**. Further inquiries can be directed to the corresponding author/s.

## Ethics statement

Ethical review and approval was not required for the study on human participants in accordance with the local legislation and institutional requirements. Written informed consent for participation was not required for this study in accordance with the national legislation and the institutional requirements.

## Author contributions

YL conceived the conception and designed the study. BT and XZ contributed to the writing of the manuscript. YL and XY performed the data analyses. RL and WW contributed to the language modification, manuscript review and reference search. XZ and YL helped with the final revision of the article. All authors contributed to the article and approved the submitted version.

## Conflict of interest

The authors declare that the research was conducted in the absence of any commercial or financial relationships that could be construed as a potential conflict of interest.

## Publisher’s note

All claims expressed in this article are solely those of the authors and do not necessarily represent those of their affiliated organizations, or those of the publisher, the editors and the reviewers. Any product that may be evaluated in this article, or claim that may be made by its manufacturer, is not guaranteed or endorsed by the publisher.

## Abbreviations

HCC, Hepatocellular carcinoma; TACE, Transcatheter arterial chemoembolization; TIME, tumor immune microenvironment; TCGA, The Cancer Genome Atlas; ICGC, International Cancer Genome Consortium; ARGs, angiogenesis-related genes; GO, Gene Ontology; KEGG, Kyoto Encyclopedia of Genes and Genomes; CDF, cumulative distribution function; LASSO, least absolute shrinkage and selection operator; PCA, Principal component analysis; TMB, tumor mutation burden; ROC, receiver operating characteristic; K-M, Kaplan–Meier; AUC, area under the ROC curve; IPS, immunophenoscore; TIDE, tumor immune dysfunction and exclusion; TCIA, the cancer immunome atlas; IC50, half-maximal inhibitory concentration; GDSC, genomics of drug sensitivity in cancer; ICIs, immune checkpoint inhibitors.
