# Supplementary material for: Construction and validation of an angiogenesis-related scoring model to predict prognosis, tumor immune microenvironment and therapeutic response in hepatocellular carcinoma
Source: Front Immunol. 2022 Nov 17;13:1013248. doi: 10.3389/fimmu.2022.1013248 (PMC9712199; doi:10.3389/fimmu.2022.1013248)
Supplement: Supplementary file 1 [file DataSheet_1.docx]

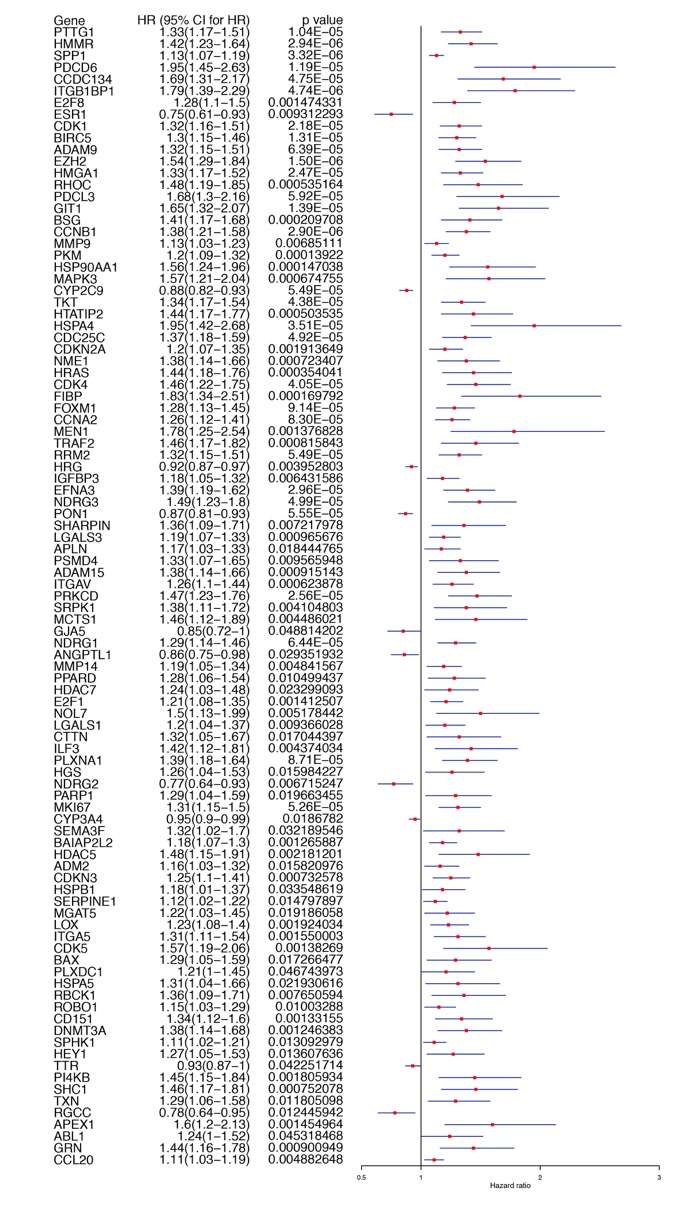


Figure S1. The HR of the identified prognostic ARGs.


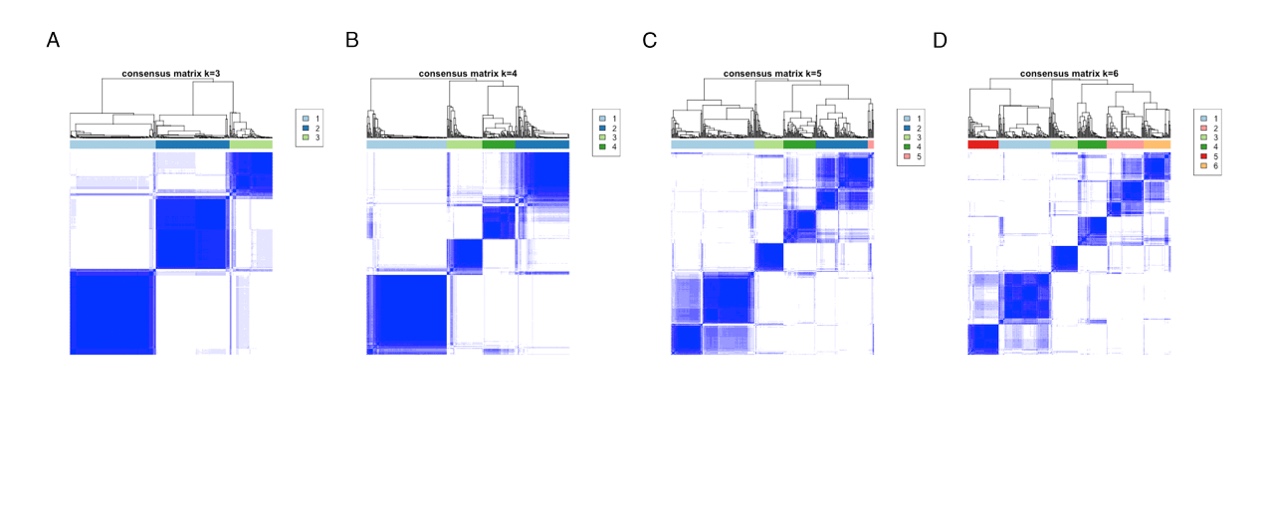


Figure S2. ﻿Consensus matrix heatmap of the clusters (k = 3-6).


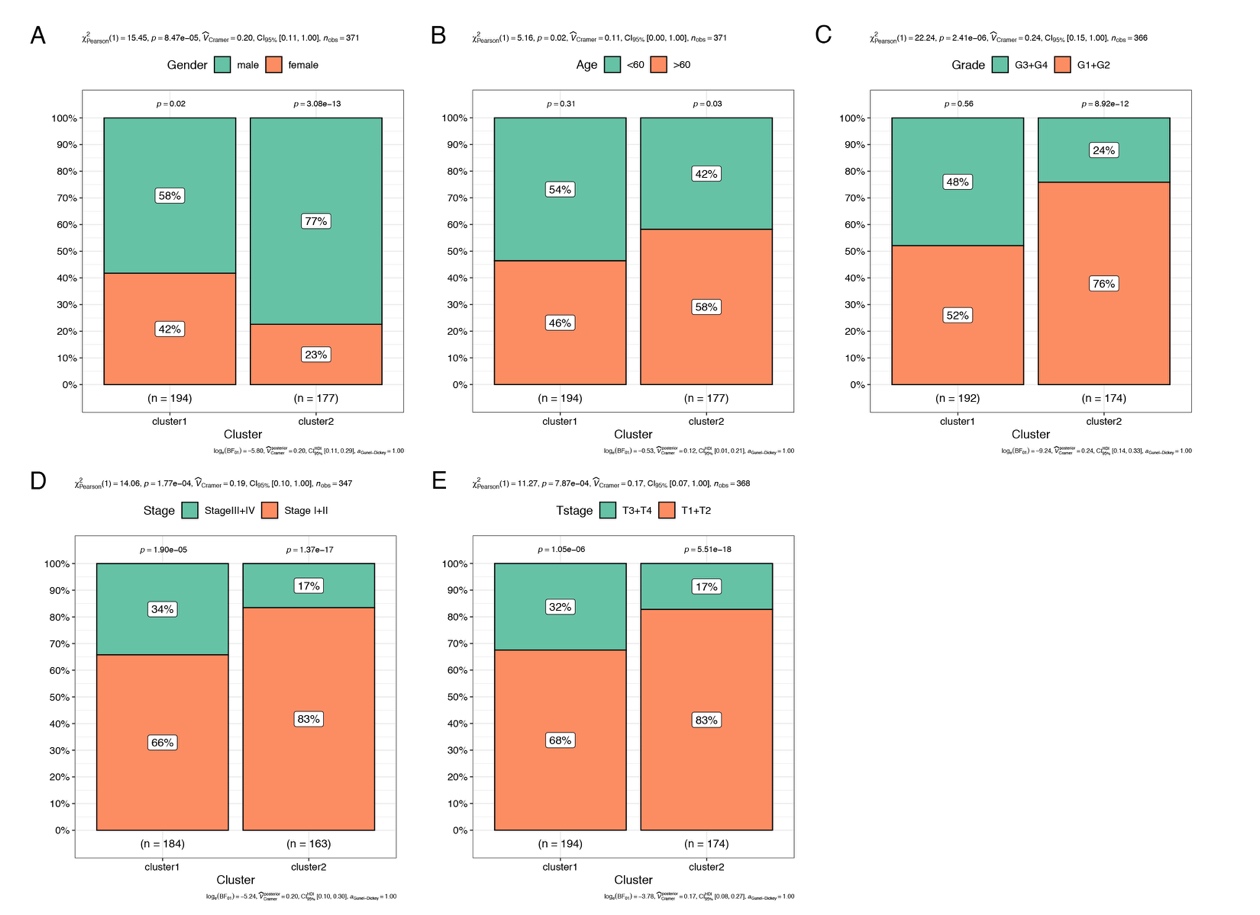


Figure S3. The correlation of clinical features between the two clusters. (A) Gender. (B) Age. (C) Histological grade. (D) Clinical stage. (E) T stage.
